# Transactions of the New Jersey Medical Society, for 1860

**Published:** 1861-07

**Authors:** 


TRANSACTIONS OF THE NEW JERSEY MEDICAL SOCIETY—For 1860, held at the City of Trenton, January, 24th and 26th, 1860; being the Ninety-Fourth Annual Meeting of the Society.

We are indebted to Recording Secretary, Wm. Pierson, Jr., M. D., for a copy of the Transactions of this sterling society, one of the oldest in the country. Its contents are valuable, and the report of the Standing Committee quite interesting.

